# Electrothermal Oxidation of Ethylene Glycol Over Co_3_O_4_

**DOI:** 10.1002/anie.1818551

**Published:** 2026-06-18

**Authors:** Adarsh Koul, Catalina Leiva‐Leroy, Moritz Lukas Krebs, Julius Ponhöfer, Jean Pascal Fandré, Anirudha Shekhawat, Harun Tüysüz, Ferdi Schüth, Martin Muhler, Wolfgang Schuhmann

**Affiliations:** ^1^ Analytical Chemistry – Center For Electrochemical Sciences (CES); Faculty of Chemistry and Biochemistry Ruhr University Bochum, Universitätsstr. 150 Bochum Germany; ^2^ Lehrstuhl für Technische Chemie, Faculty of Chemistry and Biochemistry Ruhr University Bochum, Universitätsstr. 150 Bochum Germany; ^3^ Max‐Planck‐Institut für Kohlenforschung Mülheim an der Ruhr Germany; ^4^ IMDEA Materials Institute Getafe Madrid Spain

**Keywords:** cobalt spinel, electrothermal, ethylene glycol, oxidation reaction, virtual faradaic efficiency

## Abstract

Purely electrocatalytic routes for the selective oxidation of alcohols are often limited by overpotential, mass transport, and kinetics due to multiple proton‐coupled electron‐transfer steps. We report the electrothermal oxidation of ethylene glycol (EG) over cobalt oxide (Co_3_O_4_) spinel acting simultaneously as an electrocatalyst and a solid thermocatalyst by integrating enhanced temperature and 15 bar O_2_ pressure in alkaline electrolyte at controlled current density. Enhanced EG oxidation was observed with increasing temperature from 30°C to 90°C. Glycolate and formate were the main products, while oxalate was only detected in long‐term experiments at lower current densities. Selectivity shifted from formate at low temperature, caused by electrochemical C–C cleavage, to rapid glycolate desorption at increased thermal conditions. The virtual Faradaic efficiencies (vFEs), which are the sum of all charge stored in the reaction products, reached 180% at 90°C, confirming that thermal oxidation by O_2_ at high pressure contributes to an additional oxidative electron transfer at the solid‐electrolyte interface beyond the applied electrochemical potential. These findings demonstrate that superimposing thermal and electrochemical driving forces boosts performance and enables control over reaction pathways. The approach establishes a conceptual and practical framework toward synergy between electro‐ and thermocatalytic heterogeneous alcohol oxidation toward sustainable chemical transformations.

## Graphical abstract

Pressurized electrothermal oxidation of ethylene glycol enhances catalytic performance toward glycolate under mild conditions. Higher reaction rates occur at 70–90°C and 15 bar O_2_, and potential and O_2_ pressure re‐oxidize Co^2+^ to Co^3+^ at the surface. Faradaic efficiencies above 100% reveal the synergistic effects of electrochemical and thermal catalysis.

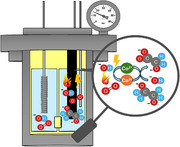

## 1 Introduction

The oxidation of alcohols to carboxylic acids is a cornerstone transformation in both synthetic chemistry and sustainable energy applications. Electrocatalysis offers a highly tunable and potentially greener alternative to thermal heterogeneous catalysis. However, production rates in electrocatalytic systems are often limited by mass transport, surface kinetics, and the overpotential required to drive the reaction, resulting in lower efficiencies compared with thermal routes [1, 2]. A promising strategy to overcome these limitations is concurrently applying temperature and enhanced oxygen pressure to boost catalytic performance. While approaches combining elevated temperature and pressure have proven effective in thermal catalysis, their systematic integration with electrocatalysis toward oxidation of alcohols remains underexplored [3, 4, 5].

Recent work has established rigorous frameworks for unifying thermal and electrochemical catalysis by treating thermal redox transformations as coupled electrochemical half‐reactions. Surendranath et al. proposed electrochemical strategies to study typical thermochemical redox reactions [6, 7, 8]. In particular, the mixed potential of a catalyst under thermal turnover can be predicted by overlaying polarization curves of the constituent oxidation/reduction half reactions. Additionally, applying external bias drives one half‐reaction and replicates the thermal rate/selectivity [9]. This framework explains both reaction rates and selectivity of products in thermal catalysis using tools of electrochemical kinetics. Peng et al. expanded this formalism, generalizing that any thermocatalytic redox process in water can be deconvoluted into electrochemical half‐reactions, thereby connecting chemical free‐energy driving forces directly to electrode potentials [10]. This equivalence of electrochemical and thermal catalysis provides the conceptual basis for electrothermal catalysis. Complementary experimental evidence from Flaherty et al. showed that thermocatalytic and electrocatalytic H_2_O_2_ synthesis exhibit overlapping kinetics and selectivity when compared at equivalent electrochemical potentials, validating the thermodynamic equivalence of chemical and electrochemical driving forces [11]. Most recently, this framework was applied to demonstrate electrochemical promotion of thermal catalysis, wherein a modest electrochemical bias perturbs the mixed potential established under purely thermal conditions, resulting in enhanced turnover rates [12]. The significance of the ethylene glycol oxidation reaction (EGOR) extends beyond energy conversion, as EG, a PET precursor, is a candidate for both direct fuel cells and polymer upcycling, underscoring the broader industrial relevance of this reaction [13]. In our previous study, we reported a mechanistic convergence between thermal and electrocatalytic oxidation of EG on Co_3_O_4_ surfaces [14]. Both pathways rely on the Co^3+^/Co^2+^ redox couple and exhibit similar surface‑bound intermediates and selectivity of products. They only differ in the re‐oxidation of the Co^2+^ sites, which is either performed thermally by O_2_ or anodically by electron transfer at the applied potential. This suggests a conceptual bridge between thermal and electrochemical mechanisms, offering a foundation for hybrid approaches.

We present electrothermal oxidation of EG based on systematic evaluation of the influence of temperature and oxygen pressure on electrocatalytic performance. By combining thermal and electrochemical driving forces, we explore whether temperature and pressure can enhance reaction rates, and observe modulations of product distribution under electrothermal conditions. and evaluate how these parameters influence energy efficiency and yield.

## 2 Results and Discussion

**FIGURE 1:**
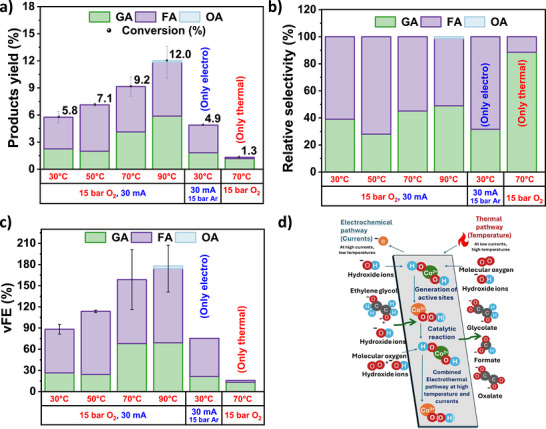
Electrothermal oxidation of ethylene glycol (EG) over Co_3_O_4_‐modified electrodes as a function of temperature for 2 h. (a) Total product yields (glycolate + formate) as a function of temperature at 15 bar O_2_. (b) Relative product selectivity trends highlight the shift from oxidation to formate at low temperature to the formation of glycolate at elevated temperature. (c) Virtual Faradaic efficiency (vFE) analysis revealing over‐unity values (> 100%) above 50 °C. The schematic regions denote electrocatalytic (vFE < 100%) and synergistic electrothermal (vFE > 100%) domains. The error bars in (a) and (c) represent standard deviations for the overall conversion and total vFE. (d) Schematic representation of a plausible reaction pathway of electrothermal oxidation of EG on Co_3_O_4_‐modified surfaces from catalyst generation to product formation to catalyst regeneration.

To investigate the effect of temperature, oxygen pressure, and current on the electrothermal oxidation of EG, we employed an electrothermal reactor using cobalt oxide spinel (Co_3_O_4_)–modified glassy carbon electrodes (loading: 1 mg cm^−2^; 2.1 cm^2^) as the working electrode. The anode compartment contained 1 M KOH and 1 M EG, while the cathode compartment was separated by a Zirfon separator and contained 1 M KOH (Figure S1). A constant current of 30 mA (current density 14.3 mA cm^−2^; total charge: 216 C) was applied in all electrochemical experiments, except for a control experiment conducted under purely thermal conditions. Experiments were performed at systematically varied conditions, and the corresponding product distributions (yields) are summarized in Figure 1a. Across all conditions, three oxidation products, glycolate (GA), formate (FA), and oxalate (OA), were detected in the electrolyte. The influence of temperature was investigated at a constant O_2_ pressure of 15 bar by performing individual experiments at increasing temperatures from 30 to 90°C. At 30°C, the conversion was ∼6%, with more than half of the products being FA. Increasing the temperature to 50°C resulted in a higher conversion (∼7.1%), indicating that thermal activation already contributes to the oxidation at relatively low temperatures. As an identical charge was passed in each experiment, the additional product formation at elevated temperature must arise from thermal contributions acting in tandem with electrocatalysis. At higher temperatures, the conversion increased further to 9.1% at 70°C and 12% at 90°C. This trend suggests a transition from charge‐transfer‐limited electrode kinetics at low temperature to thermally assisted redox turnover at higher temperature, where thermal energy increasingly facilitates Co^2+^ reoxidation alongside the applied bias.

To elucidate the individual roles of electrochemical and thermal pathways, control experiments were performed (Figure S2). Under purely electrochemical conditions (15 bar Ar, 30°C), a similar yield (∼5.2%) was obtained compared to the reaction under O_2_ (∼5.8%), indicating that the presence of O_2_ under pressure has minimal influence at low temperature and during the investigated timescale. In contrast, purely thermal conditions (70°C, 15 bar O_2_) resulted in a significantly lower product yield (∼1.5%), demonstrating that thermal oxidation alone is inefficient at these temperatures in the presence of pressurized O_2_. Similar results were observed with only non‐modified bare glassy carbon as the electrode at the same reaction conditions, demonstrating negligible contributions from homogeneous reactions (Figure S3) as suggested previously [15]. Importantly, the low activity observed at purely thermal conditions indicates that direct thermocatalytic oxidation is not a dominant pathway. Instead, thermal effects primarily facilitate restoration of the active state of the catalyst, likely by promoting reoxidation of reduced Co^2+^ species, thereby enabling enhanced EG oxidation beyond that driven solely by the applied anodic potential. Furthermore, performing the reaction under an inert atmosphere at 70°C (15 bar Ar) yielded ∼5% (Figure S2a), comparable to the electrochemical yields at 30°C, confirming that elevated temperature alone does not enhance the reaction in the absence of O_2_. Additional electrochemical experiments in the presence of O_2_, but at atmospheric pressure, resulted in similar yields (∼5.2%) (Figure S2a), highlighting the dominance of the electrochemical regime at these reaction conditions, independent of O_2_ pressure.

Notably, the yield obtained under combined electrothermal conditions (Figure 1a) at 70°C (∼9.1%) exceeds the sum of the individual contributions from electrochemical oxidation at 30°C and thermal oxidation at 70°C (∼6.5%). This clearly indicates a synergistic interaction between thermal and electrochemical pathways. At the same time, the negligible difference between experiments conducted with and without oxygen at 30°C confirms that thermal contributions remain minimal at low temperatures.

Overall, these results demonstrate that elevated temperature and O_2_ pressure cooperatively enhance catalytic turnover, establishing a synergistic electrothermal mechanism. Relative product selectivity trends of the quantified products are shown in Figure 1b. At 30°C, the electrochemically dominated reaction yields formate as the main product (∼60% selectivity). A slight increase in FA selectivity (∼70%) is observed at 50°C, consistent with conditions still dominated by electrochemical oxidation. At higher temperatures, however, FA selectivity decreases, and the reaction shifts toward glycolate (GA) formation, possibly suggesting a change in the mechanistic pathway tending more toward the thermal EG oxidation.

Control experiments conducted under purely thermal conditions (no applied current) confirm this trend, with almost exclusively GA being formed, indicating a preference for partial oxidation pathways in the absence of the electrochemical driving force. Further insight into this selectivity shift is obtained by varying the electrochemical input. At 70°C, lowering the current density to 5 mA results in a high relative selectivity toward glycolate (∼73%), indicating that under reduced electrochemical driving force, thermally assisted pathways dominate and favor partial oxidation (Figure S3b). In contrast, performing the reaction under inert atmosphere (15 bar Ar) at 70°C and higher current density (30 mA) leads to a significantly lower glycolate selectivity (∼14%) (Figure S2b), highlighting that strong electrochemical driving force promotes deeper oxidation pathways even at elevated temperatures.

Overall, these observations demonstrate that product distribution is governed by the interplay between electrochemical driving force (current density) and thermally assisted processes (enhanced at elevated temperature and in the presence of O_2_). Under predominantly electrochemical conditions, higher FA selectivity is observed, consistent with deeper oxidation pathways involving C–C bond cleavage. In contrast, when thermal contributions become more significant, selectivity shifts toward GA, indicating a preference for partial oxidation pathways.

The higher selectivity toward FA under electrochemical conditions is consistent with previous reports on alcohol electrooxidation [16], where applied potential promotes deeper oxidation and C–C bond cleavage. However, direct identification of specific cobalt oxidation states under the present electrothermal conditions is not possible, as in situ spectroscopic measurements under high‐temperature and high‐pressure conditions inside the autoclave are not possible. Therefore, the role of highly oxidized cobalt species is considered a plausible interpretation based on literature and the current experimental evidence. Under electrothermal conditions, the increased formation of GA can be rationalized by a stronger contribution from thermally assisted oxidation pathways involving molecular oxygen. In such systems, O_2_ participates in catalyst regeneration and subsequent chemical oxidation steps, which have been shown to favor partial oxidation products [17]. This contribution becomes more pronounced at elevated temperatures due to enhanced oxygen activation and turnover rates [14]. As shown in Figure 1d, regeneration of active Co species at lower temperatures is primarily governed by electrochemical oxidation at the applied potential. With increasing temperature, molecular oxygen increasingly contributes to catalyst regeneration, resulting in a combined electrochemical and thermally assisted redox cycle. Importantly, these pathways are not mutually exclusive, and electrochemical oxidation remains operative even at elevated temperatures.

To compare the efficiency of the electrothermal process, we introduce the virtual Faradaic efficiency (vFE), which accounts for both electrochemical and thermal contributions to the transferred charge and is the sum of all charge required for the formation of all products during EG oxidation (Figure 1c). Thus, vFE values exceeding 100% reflect additional chemically driven oxidation events that are not directly coupled with electron flow through the external circuit. At 30°C, vFE was ∼90%, slightly below the ideal 100% expected from a purely electrochemical process, most likely due to charge consumption by carbonate formation due to EG overoxidation. This hypothesis was supported by the carbonate peaks in the post‐reaction XRD pattern of the Co_3_O_4_ catalyst (Figure S4). At 50°C, vFE exceeded 100% (∼115%), showing that thermal energy contributes additional charge transfer beyond the charge supplied via the external electrical circuit.

This over‐unity efficiency arises because Co^2+^ centers are oxidized not only by electron transfer to the electrode, but also by activated molecular O_2_, which acts as a secondary oxidant at elevated temperature and pressure. Such self‐sustained redox cycling mirrors the mixed‐potential behavior [9, 10]. At even higher temperatures, vFE increased substantially, reaching ∼150% at 70°C and ∼180% at 90°C.

**FIGURE 2:**
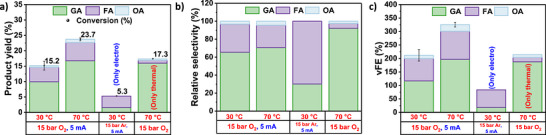
(a) Product yields, (b) product selectivity, and (c) virtual Faradaic efficiencies for experiments performed at a current of 5 mA for 12 h at 30°C and 70°C. (green—FA; violet—GA; light blue—oxalic acid). The error bars in (a) and (c) represent standard deviations for the overall conversion and total vFE.

Electrothermal reactions were also performed over longer time periods to amplify the thermal effects; nonetheless, passing the same charge as in the previous experiments by decreasing the applied current for keeping the electrochemical effects consistent. Figure 2a shows that longer reaction time (12 h) leads to almost tripled conversion compared to the short‐term experiment for the same electrocatalytically transferred charge. Interestingly, oxalate (OA) was also detected although at very low amounts. In comparison, the control experiment, that is, the purely electrochemical oxidation performed at 30°C in Ar, showed similar yields relative to the control experiment at a shorter time scale, indicating that oxygen pressure is necessary to invoke thermal oxidation. At 70°C, both higher products yield (∼24%) and higher selectivity to GA and OA were obtained compared with reactions at 30°C again indicating that thermal oxidation favors oxidation avoiding C‐C splitting pathways (Figure 2b). The observed effects cannot be attributed to purely thermal oxidation of EG, as the standalone control thermal reaction exhibits a lower yield (∼17%) at 70°C (Figure 2a), indicating that potential as well as oxygen‐assisted Co redox cycling is essential. This demonstrates that both electrochemical and thermal pathways contribute to the overall reaction under these conditions. Notably, at 30°C the vFE exceeded 200%, more than double as the purely electrochemical reaction, strongly suggesting a dominant role of O_2_ pressure in oxidizing Co species at lower currents and longer timescales. Increasing the temperature to 70°C leads to an even higher vFE of approximately 330%, where both oxygen‐assisted cobalt redox cycling and electrochemical oxidation synergistically act together with stronger contributions from the thermal pathway. The vFE for the control thermal reaction remained close to 200%, lower than in the combined electrothermal reaction. These results demonstrate a pronounced synergy between electrocatalytic and thermocatalytic pathways. The 12 h experiments also highlight the role of O_2_ pressure in the observed yield and selectivity increase to GA, as at identical charge transfer but extended reaction time, the GA yield increased from 1.7% to 15% and the selectivity from 40% to 65% at 30°C and 15 bar O_2_ in the short and long‐term reactions, respectively.

Post‐electrolysis characterization confirmed the structural robustness of Co_3_O_4_. The spinel phase remained intact with only minor surface cracking and agglomeration observed by SEM. Similar post‐characterization under harsher electrochemical and purely thermal conditions reported previously likewise revealed no major structural degradation apart from slight particle coarsening [14]. XRD showed the emergence of weak carbonate‐associated reflections, consistent with formate/carbonate intermediates formed during EG oxidation (Figure S4). Figure S5a shows voltammograms for ethylene glycol oxidation in alkaline electrolyte at different temperatures and gas pressures. The LSVs have comparable shapes, and as expected, the current response increases with temperature, reflecting enhanced reaction rates and mass transport.

Carbon balances based on quantified liquid products exceed 90% in most cases (Table S1). While carbonate formation is indicated by the vFE and XRD, its accurate quantification is challenging due to background carbonate present in KOH and the relatively low conversion levels. Therefore, yields and virtual Faradaic efficiencies were calculated consistently only based on the detected liquid products. ICP‐MS analysis (Table S2) of the post‐reaction electrolyte detected only trace amounts of dissolved cobalt (78 ppb), suggesting that homogeneous contributions from leached Co species are negligible under the used conditions. Interestingly, the highest leaching was observed for the electrochemical reaction performed in air, with a leaching of 110 ppb, implying that elevated gas pressure may also prevent leaching. Collectively, these results suggest three mechanistically overlapping regimes, namely a low‐temperature predominantly electrocatalytic EG oxidation at high current densities, a low‐temperature regime at lower current densities, where the effect of O_2_ pressure is noticeable at longer reaction times, and a cooperative thermal and electrochemical reaction at 50–90°C.

The experimental observations in this research work and previously reported studies on alcohol oxidation suggest a plausible mechanistic convergence in thermal and electrochemical pathways involving redox transformations at the Co_3_O_4_ surface. Under alkaline conditions, ethylene glycol can interact with cobalt surface sites, leading to the formation of reactive surface‐bound species that undergo oxidation through proton‐coupled electron transfer processes [18, 19]. Under electrochemical conditions, these processes are driven by the electron transfer at the electrode, whereas in the electrothermal setup the available O_2_ at high partial pressure may also function as a chemical oxidant, facilitating the reoxidation of reduced cobalt species. In our previous article, we showed that both the applied potential and dissolved O_2_ sustain the population of high‐valent Co^3+^ centers required for EG oxidation. Following initial oxidation steps, intermediate species may undergo further transformations in solution or upon re‐adsorption, leading to the formation of glycolate, formate, and oxalate. The relative contribution of these pathways is reflected in the observed product distribution under different reaction conditions.

Overall, these observations are consistent with a catalytic cycle involving the redox interplay of cobalt surface species, sustained by both electrochemical and thermally assisted processes. However, it should be emphasized that the proposed pathway represents a plausible interpretation based on experimental trends and prior literature, as direct spectroscopic identification of intermediates in the current experimental setup is not possible.

## 3 Conclusions

EG oxidation under electrothermal conditions proceeds with high selectivity, yielding only GA and FA as products at increasing yields with rising temperature at constant O_2_ pressure. Importantly, the product distribution is dependent on the balance between thermal and electrochemical contributions, i.e., at lower temperatures, the process is dominated by electrochemical pathways, favoring C–C cleavage and formate production, whereas at higher temperatures thermal re‐oxidation of the Co^2+^ sites by O_2_ becomes more prominent, leading to a shift of the product distribution to glycolate. We introduce the concept of virtual Faradaic efficiencies (vFE) as a measure for electrothermal synergy and for comparing reactions with the charge stored in all formed products exceeding 100% of the charge flowing through the external electrical circuit. O_2_ pressure and temperature can act as chemical driving forces that supplement the applied potential. Mechanistically, the synergy between thermal and electrochemical oxidation of Co^2+^ enhances the steady‐state availability of Co^3+^ sites, thereby accelerating overall EG oxidation. These results constitute direct experimental evidence of electrothermal coupling in a liquid‐phase oxidation reaction. From an application perspective, such electrothermal systems could be integrated into industrial environments where oxygen and low‐grade heat are readily available as by‐products, enabling more efficient utilization of both electrical and thermal energy inputs. Further insight into the nature of surface intermediates and elementary steps under electrothermal conditions would require in situ or operando spectroscopic investigations at elevated temperature and pressure which is presently not possible for the current setup.

## Author Contributions

**Adarsh Koul**: investigation, writing – original draft, writing – review and editing, formal analysis. **Catalina Leiva‐Leroy**: investigation, writing – original draft, writing – review and editing, formal analysis. **Moritz Lukas Krebs**: investigation, writing – review and editing, methodology, data curation. **Julius Ponhöfer**: investigation, formal analysis. **Jean Pascal Fandré**: investigation, writing – review and editing. **Anirudha Shekhawat**: investigation. **Harun Tüysüz**: writing – review and editing, funding acquisition, resources, supervision, data curation. **Ferdi Schüth**: conceptualization, methodology, writing – review and editing, supervision, resources, project administration, validation, funding acquisition. **Martin Muhler**: conceptualization, validation, writing – review and editing, writing – original draft, project administration, supervision, resources, funding acquisition. **Wolfgang Schuhmann**: conceptualization, funding acquisition, writing – original draft, writing – review and editing, validation, methodology, project administration, supervision, resources.

## Conflicts of Interest

The authors declare no conflicts of interest.

## Supporting information




**Supporting File**: The authors have cited additional references within the Supporting Information [20, 21].

## Data Availability

The data that support the findings of this study are openly available in Zenodo at https://zenodo.org/, reference number 10.5281/zenodo.18017695.
