# Supplementary material for: Electrothermal Oxidation of Ethylene Glycol Over Co3O4
Source: Angew Chem Int Ed Engl. 2026 Jun 18;65(32):e1818551. doi: 10.1002/anie.1818551 (PMC13427236; doi:10.1002/anie.1818551)
Supplement: Supplementary file 1 — Supporting File: The authors have cited additional references within the Supporting Information [20, 21]. [file ANIE-65-e1818551-s001.docx]

**Supporting information**

Electrothermal Oxidation of Ethylene Glycol over Co_3_O_4_

Adarsh Koul^†[a]^, Catalina Leiva-Leroy^†[b]^, Moritz Lukas Krebs^†[c]^, Julius Ponhöfer ^[c]^, Jean Pascal Fandré^[c]^, Anirudha Shekhawat^[a]^, Harun Tüysüz^[d]^, Ferdi Schüth^[c]^, Martin Muhler^[b]^, Wolfgang Schuhmann*^[a]^

**Catalyst synthesis**

The SBA-15 hard template was prepared following a reported procedure.^[1]^ For the preparation of two-dimensional ordered mesoporous Co_3_O_4_, Co(NO_3_)_2_·6H_2_O (ACS reagent, ≥98%) was first dissolved in ethanol (absolute for analysis EMSURE ACS, ISO, Reag. Ph Eur; Merck) to obtain a 0.8 M solution. This precursor solution was impregnated into the SBA-15 by first mixing 70 vol% of the solution with the SBA-15 and stirring at 500 RPM for one hour at room temperature in a 250 mL PP beaker. The resulting suspension was dried at 40 °C and then calcined at 250°C for 4 h using a heating rate of 5°C min^-1^. The calcined powder was subsequently impregnated again with the remaining 0.8 M precursor solution and stirred at 250 rpm for 1 h, followed by drying at 40°C. A second calcination step was carried out at 250°C for 4 h (5°C min^-1^ heating rate), after which the temperature was further increased to 500°C at a slower rate of 2°C min^-1^ and held for 6 h. To remove the SBA-15 template, the calcined material was treated with 20 mL of 2 M NaOH (≥98%, pellets, GPR RECTAPUR) solution at 80°C and shaken. The suspension was decanted, replenished with fresh NaOH solution, and left overnight at 80°C. The resulting dispersion was repeatedly washed with deionized water and centrifuged until a neutral pH was reached for the supernatant. Finally, the sediment was dried in an oven at 80°C overnight to yield the catalyst powder.

**Electrochemical setup and measurement**

Electrochemical experiments were conducted in a custom-built autoclave (Figure S1) which was a modified and improved version of the initial system used in ^[2]^. The setup could sustain temperatures of up to 473 K and pressures of up to 100 bar. Pressure was monitored using a JUMO dTrans p30 sensor, while temperature was recorded with a type K thermocouple housed in a Teflon sleeve. The autoclave exterior is constructed from stainless steel (1.4571), and the interior is lined with Teflon. Sealing was achieved with PEEK and Teflon components. Platinum wires provided electrical contacts.

The working electrode was prepared by drop-coating Co_3_O_4_ catalyst ink (loading: 1 mg cm^-2^) onto both sides of a glassy carbon plate, giving a total exposed catalyst area of 2 cm^2^. The catalyst ink was prepared by dissolving 2 mg of catalyst in 96 µL ethanol and 4 µL Nafion. A Pt coil (geometric area ≈ 14 cm^2^) served as counter electrode, while a third Pt wire placed in the anolyte compartment acted as pseudo-reference electrode. Anode and cathode compartments were separated by a Zirfon Perl UTF 500 diaphragm. The catholyte consisted of 1 M KOH, and the anolyte contained 1 M KOH with 1 M ethylene glycol (6 mL per compartment). Magnetic stirring was applied in both compartments to enhance mass transport. After electrolyte filling and electrode placement, the autoclave was sealed and pressurized with O_2_ to the desired pressure.

Electrochemical measurements were carried out using a Gamry Interface 1010E poten­tiostat. Prior to testing, open-circuit voltage and uncompensated resistance were determined to verify system stability, yielding solution resistances between 1 and 2 Ω. Three consecutive cyclic voltammograms were then recorded in the potential range of 0.3–1.0 V vs the Pt pseudo-reference at a scan rate of 50 mV s^-1^. Chronopotentiometry was performed at a current of 30 mA for 2 h. After completion, the autoclave was cooled in a water bath before sampling the electrolytes for HPLC analysis.

**
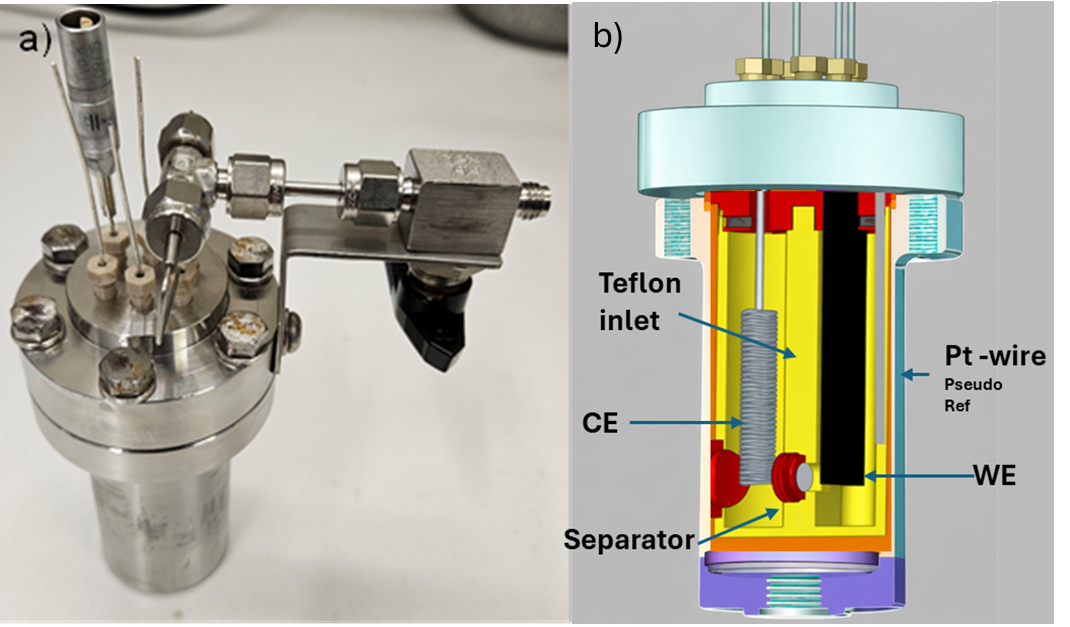
**

*Figure S1* Schematic illustration of the autoclave setup. (a) Schematic image of the interior components in the pressurized electrothermal reactor, including the Pt coil counter electrode (CE), Co_3_O_4_-modified glassy carbon electrodes as working electrode (WE) and a Zirfon Perl UTF 500 diaphragm separator. All parts are surrounded by a PTFE liner. (b) Photograph of the reaction autoclave.

All potentials are referenced to a Pt wire pseudo-reference placed in the anolyte com­partment. Because a pseudo-reference does not provide a stable potential in alkaline electrolyte and can drift with dissolved species (e.g., dissolved O_2_), the absolute potential values are not interpreted further. Instead, the discussion is focused on a qualitative current–potential behavior from CVs and on product formation after passing identical charges during chronopotentiometry experiments.

**HPLC characterization**

1 mL of the samples (containing KOH. ethylene glycol, formate, glycolate, oxalate and carbonates) were taken after each electrolysis and acidified with sulfuric acid for analysis by means of high-performance liquid chromatography (HPLC) (Azura, Knauer). An Aminex HPX-87H (Biorad) column was utilized to determine the concentrations of ethylene glycol (EG), glycolic acid (GA), oxalic acid (OA) and formic acid (FA).

**Thermal and electrochemical catalytic calculations.**

Conversion (X) related to the measured products in the liquid phase was calculated as:

$$X\left( \% \right)=\frac{n_{\mathrm{EG}_{\mathrm{Initial}}}-n_{\mathrm{EG}_{\mathrm{Final}}}}{n_{\mathrm{EG}_{\mathrm{Final}}}+n_{\mathrm{GA}}+\frac{1}{2}n_{\mathrm{FA}}+n_{\mathrm{OA}}}*100\%$$

Where $n_{i}$ refers to moles of reactant or product.

While for the calculation of the carbon balance (CB) was:

$$\mathrm{CB}\left( \% \right)=\frac{2*n_{\mathrm{EG}_{\mathrm{Final}}}+2*n_{\mathrm{GA}}+n_{\mathrm{FA}}+2*n_{\mathrm{OA}}}{2*n_{\mathrm{EG}_{\mathrm{Initial}}}}*100\%$$

The relative selectivity ($S_{i}$) toward individual liquid products was calculated as follows:

$$S_{\mathrm{GA}}\left( \% \right)=\frac{n_{\mathrm{GA}}}{n_{\mathrm{GA}}+\frac{1}{2}n_{\mathrm{FA}}+n_{\mathrm{OA}}}*100\%$$

$$S_{\mathrm{FA}} \left( \% \right)= \frac{n_{\mathrm{FA}}}{n_{\mathrm{GA}}+\frac{1}{2}n_{\mathrm{FA}}+n_{\mathrm{OA}}}*100\%$$

$$S_{\mathrm{OA}} \left( \% \right)= \frac{n_{\mathrm{OA}}}{n_{\mathrm{GA}}+\frac{1}{2}n_{\mathrm{FA}}+n_{\mathrm{OA}}}*100\%$$

Yields ($Y_{i}$) towards individual products were calculated as follows:

$$Y_{\mathrm{GA}}\left( \% \right)=Selectivity GA \left( \% \right)*Conversion(\%)$$

$$Y_{\mathrm{FA}} \left( \% \right)=Selectivity FA \left( \% \right)*Conversion(\%)$$

$$Y_{\mathrm{OA}} \left( \% \right)=Selectivity OA \left( \% \right)*Conversion(\%)$$

The virtual Faradaic efficiency (FE) for each product formed during ethylene glycol oxidation was calculated as:

$$\mathrm{FE}\left( \% \right)= \frac{n.z.F}{Q}*100\%$$

where n is the number of moles of the respective product (determined by HPLC), z is the number of electrons transferred per mole of that product, F is the Faraday constant (96,485 C mol^-1^), and Q is the total charge passed. Chronopotentiometry was performed at 30 mA for 2 h, corresponding to a fixed charge input of 216 C. Therefore, the reported values represent *virtual Faradaic efficiencies*, enabling direct comparison of product distribution between different conditions and different pathways, namely electrochemical and thermal, based on equal charge.

***Figure S2*** Electrothermal oxidation of ethylene glycol (EG) over Co_3_O_4_-modified electrodes as a function of current and temperature. (a) Total product yields (glycolate + formate) show no effect of pressure at 30 °C. (b) Relative product selectivity showing mainly formate generation in electrochemical regime. (c) Virtual Faradaic efficiency (vFE) relative to all measurements, denoting the control of electrochemistry under anaerobic conditions.

***Figure S3*** Electrothermal oxidation of ethylene glycol (EG) over Co_3_O_4_-modified electrodes as a function of current and catalysis. (a) Total product yields (glycolate + formate) show low conversion of the homogeneously catalyzed reaction (glassy carbon as electrode). (b) Relative product selectivity shows mainly glycolate generation in the thermal regime. (c) Virtual Faradaic efficiency (vFE) denotes the control of thermal catalysis when performing the reaction at low currents.

***Table S1.*** Experimental details, product yield and carbon balance for all the performed experiments as function of the detected liquid products.

| **Experiment** | **Temp (°C)** | **Gas  (15 bar)** | **Current**  **(mA)** | **Time (h)** | **X (%)** | **Y_GA_**  **(%)** | **Y_FA_**  **(%)** | **Y_OA_**  **(%)** | **CB (%)** |
| --- | --- | --- | --- | --- | --- | --- | --- | --- | --- |
| ***Short-term (2 h)*** |  |  |  |  |  |  |  |  |  |
| **EXP1** | 30 | O_2_ | 30 | 2 | 5.3 | 1.7 | 3.6 | - | 92 |
| **EXP2** | 30 | O_2_ | 30 | 2 | 6.2 | 2.8 | 3.4 | - | 93 |
| **EXP3** | 50 | O_2_ | 30 | 2 | 7.0 | 1.3 | 5.6 | - | 90 |
| **EXP4** | 50 | O_2_ | 30 | 2 | 7.3 | 2.7 | 4.6 | - | 95 |
| **EXP5** | 70 | O_2_ | 30 | 2 | 8.4 | 3.2 | 5.1 | - | 95 |
| **EXP6** | 70 | O_2_ | 30 | 2 | 9.9 | 5.0 | 4.9 | - | 96 |
| **EXP7** | 70 | O_2_ | 5 | 2 | 3.4 | 2.5 | 0.9 | - | 97 |
| **EXP8** | 70 | O_2_ | - | 2 | 1.5 | 1.3 | 0.2 | - | 98 |
| **EXP9** | 70 | O_2_ | glassy carbon | 2 | 1.5 | 1.3 | 0.1 | - | 95 |
| **EXP10** | 70 | O_2_ | - | 2 | 1.1 | 1.0 | 0.1 | - | 95 |
| **EXP11** | 90 | O_2_ | 30 | 2 | 10.6 | 5.5 | 5.1 | - | 97 |
| **EXP12** | 90 | O_2_ | 30 | 2 | 13.1 | 6.3 | 6.8 | 0.3 | 93 |
| **EXP13** | 30 | Ar | 30 | 2 | 4.9 | 1.8 | 3.1 | - | 97 |
| **EXP14** | 30 | - | 30 | 2 | 5.1 | 1.6 | 3.5 | - | 91 |
| **EXP15** | 30 | - | 30 | 2 | 5.3 | 1.7 | 3.5 | - | 93 |
| **EXP16** | 70 | Ar | 30 | 2 | 5.0 | 0.7 | 4.3 | - | 90 |
| ***Long-term (12 h)*** |  |  |  |  |  |  |  |  |  |
| **OEXP1** | 30 | O_2_ | 5 | 12 | 16.2 | 10.5 | 5.1 | 0.6 | 98 |
| **OEXP2** | 30 | O_2_ | 5 | 12 | 14.2 | 9.4 | 4.4 | 0.4 | 88 |
| **OEXP3** | 70 | O_2_ | 5 | 12 | 23.4 | 15.5 | 5.9 | 1.9 | 85 |
| **OEXP4** | 70 | O_2_ | 5 | 12 | 24.0 | 18.0 | 5.9 | 0.2 | 98 |
| **OEXP5** | 70 | O_2_ | - | 12 | 17.3 | 16.0 | 0.9 | 0.4 | 95 |
| **OEXP6** | 30 | Ar | 5 | 12 | 5.3 | 1.6 | 3.7 | - | 93 |

***Table S2.*** ICP-MS analysis of the post reaction electrolyte

| **Experiment** | **Co content (parts per billion (ppb))** | **µmol/L** | **Leached Co compared to the solid electrolyte (%)** |
| --- | --- | --- | --- |
| **30 °C, 15 bar O_2_, 30 mA, 2h** | 14.05 | 0.23 | 0.001 |
| **50 °C, 15 bar O_2_, 30 mA, 2h** | 34.61 | 0.58 | 0.003 |
| **70 °C, 15 bar O_2_, 30 mA, 2h** | 78.07 | 1.32 | 0.007 |
| **70 °C, 15 bar O_2_, 2h** | 23.33 | 0.39 | 0.002 |
| **30 °C, 30 mA, 2h** | 110.16 | 1.86 | 0.011 |
| **30 °C, 15 bar O_2_, 5 mA, 12h** | 71.04 | 1.20 | 0.007 |

**Post reaction characterization**

The diffraction pattern of the as-synthesized catalyst matches well with the characteristic reflections of the Co_3_O_4_ spinel phase, confirming the successful synthesis of phase-pure cobalt oxide. After electrolysis, the main Co_3_O_4_ reflections are preserved, indicating that the overall crystal structure remains stable under reaction conditions. In addition to the spinel reflections, new diffraction features appear at approximately 24.1°, 29.9°, and 39.2°. These can be assigned to potassium bicarbonate phases, which are plausibly formed during operation. Ethylene glycol oxidation proceeds through partially oxidized interme­diates such as formate, which can further oxidize to carbonate species. In alkaline electro­lyte, these carbonates may react with potassium ions from the supporting KOH to yield potassium bicarbonate upon drying of the electrolyte. The associated hydrogen transfer steps are consistent with proton-coupled electron transfer (PCET) pathways known for alcohol oxidation. Thus, the post-electrolysis XRD suggests the coexistence of preserved Co_3_O_4_ and in situ formed potassium bicarbonate residues.


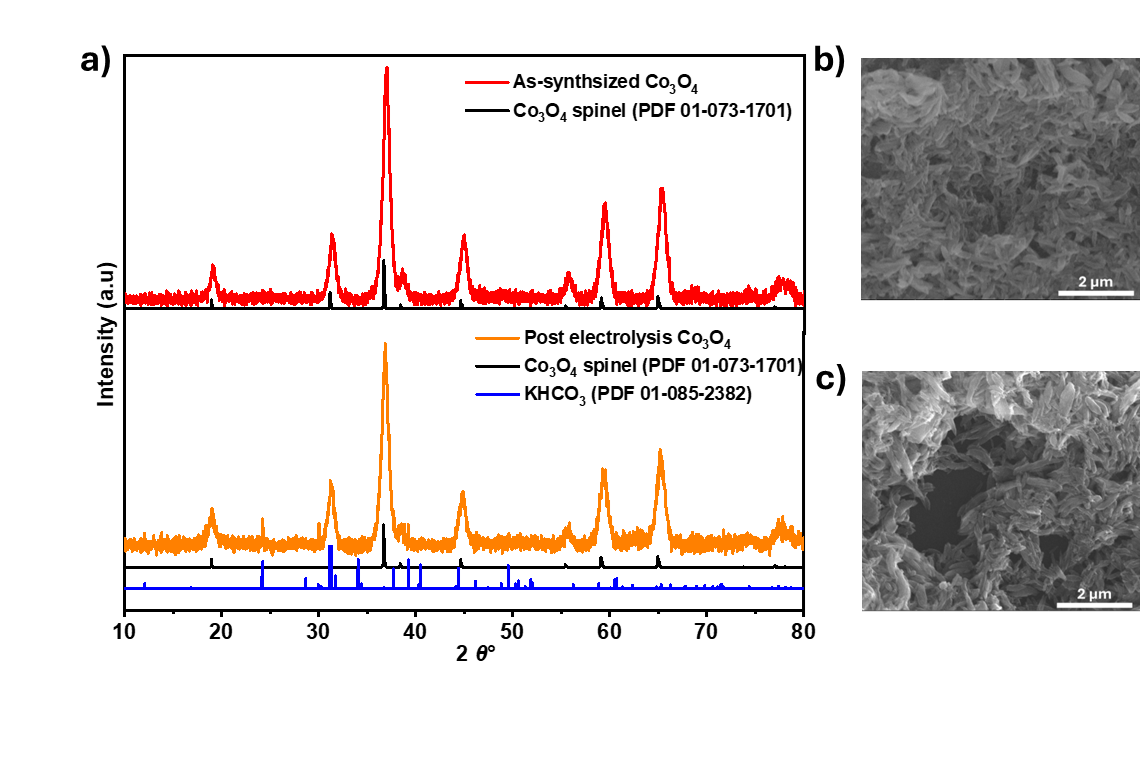


*Figure S4.* (a) Powder X-ray diffraction of the catalyst before and after reaction. Scanning electron microscopy of the (b) as synthesized and (c) post-catalysis samples.

SEM images recorded before and after electrolysis reveal preservation of the nanostruc­tured morphology of the catalyst. The Co_3_O_4_ material retains its characteristic nanowire bundles, and no significant coarsening, agglomeration, or collapse is observed. This indicates that the Co_3_O_4_ catalyst maintains both its crystallinity and nanoscale structure under the applied reaction conditions, further supporting its robustness during prolonged operation.

Figure S5a shows voltammograms (LSVs extracted from cyclic voltammograms) for ethylene glycol oxidation in alkaline electrolyte at different temperatures and gas pres­sures. The LSVs exhibit comparable overall shapes across all conditions, indicating that the underlying reaction mechanism remains consistent. As expected, the current response increases with temperature, reflecting enhanced reaction rates and mass transport.

Although variations in apparent slope are observed at higher temperatures, these are not interpreted as changes in intrinsic kinetics or overpotential. The use of a true reference electrode is not feasible under the high-temperature and high-pressure conditions of the autoclave system. Hence, potentials are referenced to a Pt wire pseudo-reference elec­trode. In alkaline electrolyte at elevated temperature and oxygen pressure, the Pt potential is influenced by multiple factors, including dissolved oxygen concentration, gas crossover, concurrent HER/OER processes, temperature effects, and the presence of ethylene glycol and its oxidation products. Consequently, apparent differences in potential cannot be reliably attributed to changes in electrode kinetics.

The LSV at 30 °C without O_2_ pressurization is shifted to higher apparent potentials compared to the 15 bar O_2_ case. This shift is not assigned to an increase in anode overpotential but is consistent with a change in the pseudo-reference potential under different gas environments. Therefore, no quantitative parameters such as onset potentials or kinetic trends are derived from these data.


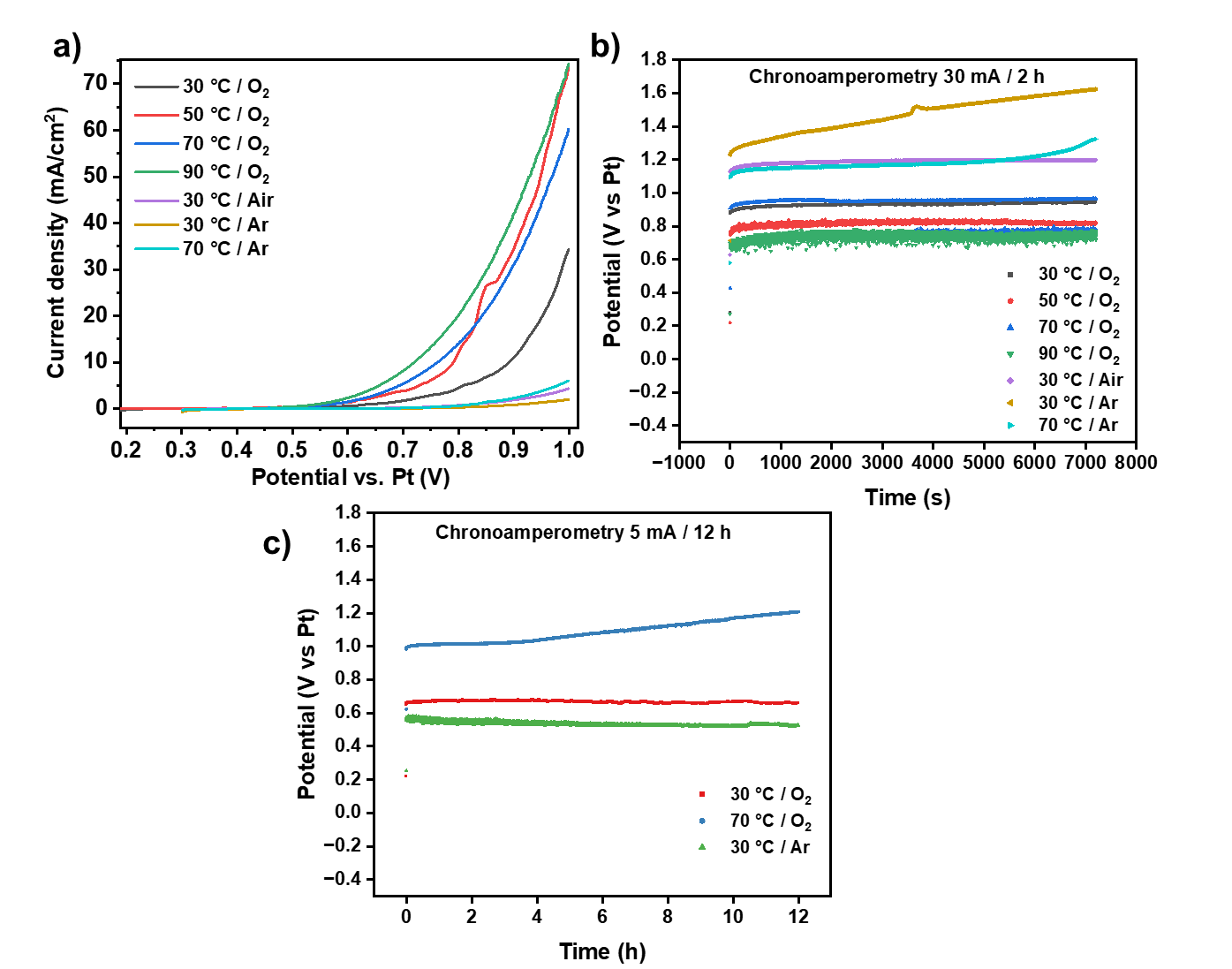


*Figure S5*. Electrochemical measurements of ethylene glycol oxidation in alkaline electrolyte. (a): Linear sweep voltammetry (LSVs) collected under the same conditions for qualitative comparison. Potentials are referenced to a Pt wire pseudo-reference in the anolyte; absolute values are not discussed owing to the potential drift of the pseudo-reference potential, and emphasis is placed on shapes and the controlled charge input. (b) and (c) Chronopotentiometry at a constant current of 30 mA for 2 h and 5 mA for 12 h, respectively. Each run in the short- and long-term measurement had the duration to ensure that an equal charge was passed for subsequent product analysis. For direct comparability, the total charge passed in both short- and long-term measurements was kept identical.

Figure S5b shows chronopotentiometric measurements at a constant current of 30 mA for 2 h. Under 15 bar O**_2_**, the recorded potentials remain relatively stable across the tempera­ture range of 30–90 °C, with only minor drift over time. In contrast, the experiment at 30 °C without O_2_ pressurization exhibits an apparent potential shift of approximately 0.3 V. However, given the instability of the pseudo-reference electrode, this shift is not interpreted as a change in intrinsic electrochemical behavior but rather as a consequence of changes in the reference potential. Figure S5c shows chronopotentiometric measure­ments over an extended period of 12 h under selected conditions. The potential remains stable over the entire measurement duration with only minor drift, indicating robust and stable electrochemical operation of the system. Differences between conditions are comparatively small and consistent with the variations shown in Figure S5b. Notably, the sustained stability over 12 h further confirms the robustness of the Co_3_O_4_ catalyst under prolonged electrothermal conditions.

Overall, electrochemical measurements are used exclusively to control the charge passed through the system under galvanostatic conditions. No quantitative interpretation of potentials, overpotentials, or reaction kinetics was made based on these data.

**References**

[1] X. Deng, K. Chen, H. Tüysüz, "Protocol for the Nanocasting Method: Preparation of Ordered Mesoporous Metal Oxides", *Chem. Mater.* **2017**, *29*, 40–52.

[2] M. L. Krebs, F. Schüth, "Electrochemical Synthesis of Nitrite and Nitrate via Cathodic Oxygen Activation in Liquefied Ammonia", *J. Am. Chem. Soc.* **2024**, *146*, 30753–30757.
